# Bayesian spatiotemporal analysis of malaria infection along an international border: Hlaingbwe Township in Myanmar and Tha-Song-Yang District in Thailand

**DOI:** 10.1186/s12936-018-2574-0

**Published:** 2018-11-16

**Authors:** Aung Minn Thway, Chawarat Rotejanaprasert, Jetsumon Sattabongkot, Siam Lawawirojwong, Aung Thi, Tin Maung Hlaing, Thiha Myint Soe, Jaranit Kaewkungwal

**Affiliations:** 1 Department of Tropical Hygiene, Faculty of Tropical Medicine, Mahidol University, Bangkok, Thailand; 2 Mahidol Vivax Research Unit, Faculty of Tropical Medicine, Mahidol University, Bangkok, Thailand; 3 Geo-Informatics and Space Technology Development Agency, Bangkok, Thailand; 4 National Malaria Control Program, Nay Pyi Taw, Myanmar; 5 Defence Services Medical Research Centre, Nay Pyi Taw, Myanmar; 6 National Malaria Control Program, Hpa-An, Myanmar

**Keywords:** Malaria, Border areas, Spatiotemporal analysis, Myanmar, Thailand

## Abstract

**Background:**

One challenge in moving towards malaria elimination is cross-border malaria infection. The implemented measures to prevent and control malaria re-introduction across the demarcation line between two countries require intensive analyses and interpretation of data from both sides, particularly in border areas, to make correct and timely decisions. Reliable maps of projected malaria distribution can help to direct intervention strategies. In this study, a Bayesian spatiotemporal analytic model was proposed for analysing and generating aggregated malaria risk maps based on the exceedance probability of malaria infection in the township-district adjacent to the border between Myanmar and Thailand. Data of individual malaria cases in Hlaingbwe Township and Tha-Song-Yang District during 2016 were extracted from routine malaria surveillance databases. Bayesian zero-inflated Poisson model was developed to identify spatial and temporal distributions and associations between malaria infections and risk factors. Maps of the descriptive statistics and posterior distribution of predicted malaria infections were also developed.

**Results:**

A similar seasonal pattern of malaria was observed in both Hlaingbwe Township and Tha-Song-Yang District during the rainy season. The analytic model indicated more cases of malaria among males and individuals aged ≥ 15 years. Mapping of aggregated risk revealed consistently high or low probabilities of malaria infection in certain village tracts or villages in interior parts of each country, with higher probability in village tracts/villages adjacent to the border in places where it could easily be crossed; some border locations with high mountains or dense forests appeared to have fewer malaria cases. The probability of becoming a hotspot cluster varied among village tracts/villages over the year, and some had close to no cases all year.

**Conclusions:**

The analytic model developed in this study could be used for assessing the probability of hotspot cluster, which would be beneficial for setting priorities and timely preventive actions in such hotspot cluster areas. This approach might help to accelerate reaching the common goal of malaria elimination in the two countries.

**Electronic supplementary material:**

The online version of this article (10.1186/s12936-018-2574-0) contains supplementary material, which is available to authorized users.

## Background

As malaria transmission continually declines, control measures will progressively rely upon precise information of the identified risk factors, as well as the capacity to characterize high-risk areas and populations for targeted interventions [1]. One challenge consistently noted in malaria elimination is cross-border malaria infection, owing to migrant populations being particularly difficult to monitor [2]. Measures to prevent and control malaria re-introduction along the border between countries require the analyses and interpretation of data from disease surveillance systems of both sides in border areas, to make correct and timely decisions [2]. Reliable maps of projected malaria distribution can help in directing intervention strategies, to optimize the use of limited human and financial resources in the areas with greatest need [3].

In this study, a spatiotemporal analytic model was proposed for assessing the malaria distribution along the border between Myanmar and Thailand, where there has been consistent malaria incidence for decades. In Myanmar, malaria has been reported in 284 out of 330 townships. The morbidity and mortality rates of malaria in Myanmar were 24.35 per 1000 population and 12.62 per 100,000 population in 1990, and 6.44 per 1000 population and 0.48 per 100,000 population in 2013, respectively. As per the World Malaria Report 2015, 32 million individuals are residing in malaria transmission zones, of whom 16% are in high-risk areas. Although the morbidity and mortality owing to malaria has been declining in Myanmar, concerns remain about population movement, especially of migrants at border areas, and the occurrence of multidrug resistance of *Plasmodium falciparum*. Such situations may arise from the fact that the Myanmar national malaria control programme (NMCP) cannot provide adequate coverage in some border areas because of the local political setting and military conflicts in those fringe areas [1, 4]. In Thailand, local malaria transmission was reported in 46 of 77 provinces, 155 of 930 districts, and 5502 of 74,956 villages in 2014; this has declined continually to 4512 villages in 2016, as reported by the Thailand NMCP. Malaria cases in Thailand have generally occurred in provinces bordering Myanmar, Cambodia and Malaysia. The challenges for NMCPs in different countries in controlling malaria situations are due to differing government policies, sociocultural and political situations, economic status, and public health infrastructure. However, timely and accurate malaria disease mapping of both sides in border areas could help with understanding the contemporaneous conditions, assessing malaria transmission patterns, and conducting objective attempts at situation management. This approach could yield more precise data for local disease control units to, for example, assess malaria management actions involving the activities of rapid response teams and village health volunteers, or enforce mosquito control and drug allocation [1, 5].

The specific aim of this study was to develop and propose a spatiotemporal analytic model for assessing the malaria situation along the border between Myanmar and Thailand, using surveillance data from Hlaingbwe Township in Myanmar and Tha-Song-Yang District in Thailand. Based on the model, the exceedance probability of malaria incidence in space and time and the effect size of demographic factors associated with malaria infections in the two countries were explored. In addition, the hierarchical modelling framework [3, 6–8] used for the analysis of malaria cases was used to identify spatiotemporal hot-spot clustering of malaria cases in each country.

## Methods

### Study area and settings

**Fig. 1:**
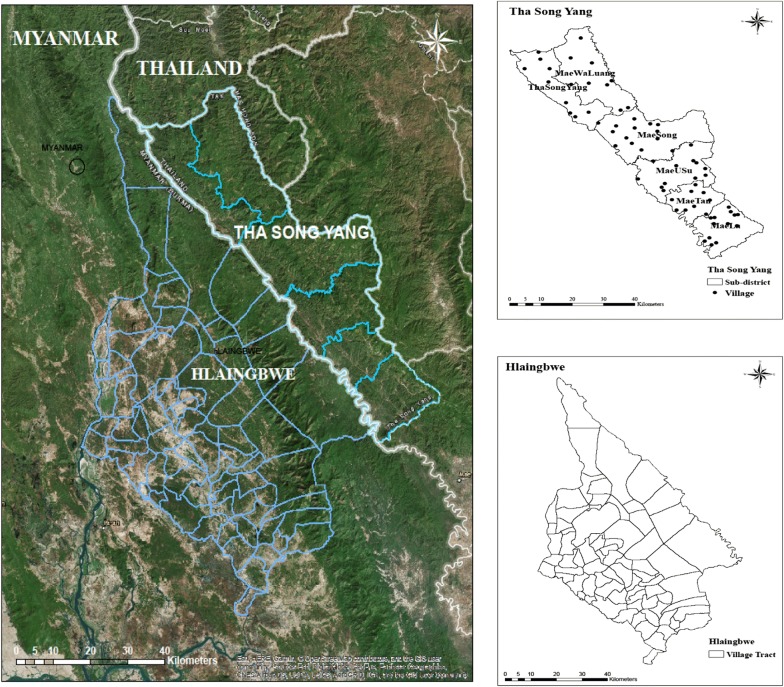
Map of the study area

The study was conducted in Hlaingbwe Township in Myanmar and Tha-Song-Yang District in Thailand. Hlaingbwe Township is the third largest township of Kayin State in Myanmar, with a population of 265,883. The area of the township is 4329.8 km^2^, and it is sub-divided into 75 village tracts. Tha-Song-Yang District is situated in Tak Province of Thailand, with a population of 61,161. The area of the district is 1920.38 km^2^, and it has 6 sub-districts with a total of 66 villages (Fig. 1). The names of the village tracts and villages in both Tha-Song-Yang and Hlaingbwe are described in the supplemental material (Additional file 1: Table S1, Additional file 2: Table S2). Malaria cases were identified as patients who were diagnosed with malaria, with either *P. falciparum*, *Plasmodium vivax,* or mixed infection, using microscopy or rapid diagnostic test.

### Data sources

Data of individual malaria cases was extracted from the routine paper-based surveillance database of the Myanmar NMCP and from Thailand’s national electronic Malaria Information System (eMIS). The malaria data used in this study included malaria cases reported at malaria clinics in both countries during the period of January to December 2016. Population and other demographic parameters for Thailand were obtained from the Statistical Yearbook Thailand 2016 [9]. For Myanmar, the information was obtained from the 2014 Myanmar Population and Housing Census published by the Department of Population, Ministry of Immigration and Population [10].

### Statistical analytic models

The Bayesian spatiotemporal zero-inflated Poisson (ZIP) model was proposed to assess the probability of disease-clustering areas. The ZIP was a proper model in this case because the counting data of malaria cases in all village tracts/villages over 12 months in the study areas had excess zeros. Some other studies also used the ZIP model in developing risk maps, for example, use of the ZIP model for mapping the malaria vector sporozoite rate [11, 12] or mapping of schistosomiasis [13], with inference made using the MCMC approach. Suitable mapping will likely be obtained with selection of a better model, which will help in evaluating risk areas.

Spatiotemporal ZIP regression model with the Bayesian approach [14–16] were assembled using WinBUGS software, version 1.4.3 (MRC Biostatistics Unit, Cambridge, UK). The monthly number of reported cases of *P. vivax*, *P. falciparum*, and mixed malaria infection (January to December 2016) in each village and village tract of Tha-Song-Yang and Hlaingbwe were analysed. An assumption was set such that the case counts are independently distributed Poisson variates. With predictors in the model (Xs) of age, gender and the interaction between age and gender for each case, the Poisson regression model was:

$$ Y_{ij} \sim Poisson(\mu_{ij} ) $$

$$ { \log } \left( {\mu_{ij} } \right) = { \log } \left( {{\text{E}}_{ij} } \right) + { \log }\left( {\theta_{ij} } \right), $$

$$\begin{aligned} \log \left( {\mu_{ij} } \right) & = \log \left( {E_{ij} } \right) + \alpha + \beta_{1} X_{ijAge}  \beta_{2} X_{ijSex} \\ & \quad + \beta_{3} X_{ijAge*Sex} + \psi_{i} + \varphi_{j} + \delta_{ij} .\end{aligned}  $$

To estimate counts over regions and time periods a form of indirect standardization, standardized incidence rate (SIR), was used; the expected rates were computed as ${\text{E}}\__{ij} = n_{ij} ( \sum _{i} \sum _{j} y_{ij} / \sum _{i} \sum _{j} n_{ij} )$ where *y*_*ij*_ is the disease count and *n*_*ij*_ is the population in the i–jth space–time unit. Although other more complex standardizations, such as stratification of the population could be pursued, such population characteristics were not available in the study databases. The above calculation was thus used to estimate the expected rates, which also represent the population offset for each space–time unit

For the excess zero counts, a spatiotemporal ZIP mixture model can be defined as:

$$ {\text{P}}\left( {Y_{ij} = y_{ij} } \right) \, = \left\{ {\begin{array}{l} {\omega_{ij} + \left( {1 - \omega_{ij} } \right)e^{{ - \mu_{ij} }} ,  \quad y_{ij} = 0} \\ {\frac{{\left( {1 - \omega_{ij} } \right)e^{{ - \mu_{ij} }} \mu_{ij}^{{y_{ij} }} }}{{y_{ij} }},     \quad \quad \quad \; \; y_{ij} > 0;} \\ \end{array} } \right. $$

$$ \log \left({\mu_{ij} } \right) = \log \left({{\text{E}}_{ij} } \right) + { \log }(\theta_{ij}) $$

$$\begin{aligned} \log \left({\mu_{ij} } \right) & = \log \left({E_{ij} } \right) + \alpha + \beta_{1} X_{ijAge} + \beta_{2} X_{ijSex} \\ & \quad + \beta_{3} X_{ijAge*Sex} + \psi_{i} + \varphi_{j} + \delta_{ij} , \end{aligned}$$

where *ω*_*ij*_ is the probability of a Bernoulli zero in village or village tract *i* in month *j* and $1 - \omega_{ij}$ is the probability of a Poisson count in village or village tract *i* in month *j*, either zero or non-zero. The beta distribution was specified for Bernoulli zero and the Poisson distribution for Poisson count.

In the model, Y_*ij*_ is the observed number of cases in village or village tract *i* in month *j*, and E_*ij*_ is the expected monthly number of cases in village or village tract *i*, which does not change by month because the population is considered static and acting as an offset. *θ*_*ij*_ is the relative risk, the parameter α is the intercept, and $\beta_{1} ,\; \beta_{2} ,\;\beta_{3}$ are the vector of coefficients for the covariates *X*_*ijAge*_, *X*_*ijSex*_, and $X_{ijAge*Sex}$; ψ_*i*_ is the spatial variability and *φ*_*j*_ is the temporal effect for each month. The spatiotemporal component δ_*ij*_ is the space and time main effect, that is, space–time clusters of risk. The shared interaction term δ_it_ gives an exchangeable hierarchical structure, i.e., *δ*_*it*_ ∼ *N*(0, $\tau_{\delta }^{ - 1}$) with a constant variance. The first-order random walk process RW(1) drives the temporal effect *φ*_*t*_ and in the random walk process, the variability of the previous month’s influence on each month except for the first one. The spatial, temporal, and space–time random effects have a uniform prior distribution for their precisions.

The spatial variability has two components: the unstructured random effect v_*i*_ with a mean of zero and precision $\tau_{\nu }^{ - 1}$, and the spatially structured random effect u_*i*_ with a mean of zero and precision $\tau_{\mu }^{ - 1}$. The spatially structured random effect is specified by a conditional autoregressive prior structure. Spatial relationships between villages or village tracts were determined using an adjacency weights matrix. If two villages or village tracts share a border, a weight of 1 is assigned whereas if they do not, the weight is 0. A normal prior distribution is specified for the coefficient whereas a flat prior distribution is specified for the intercept. The uniform prior distributions for the precisions (inverses of the variances) are specified for the unstructured and spatially structured random effects.

Markov chain Monte Carlo (MCMC) simulation techniques [17, 18] were also used to estimate the model parameters, and two chain-samplers with a burn-in of 20,000 iterations were performed. Thinning was used to lessen the autocorrelation level of the main parameters.

The deviance information criterion (DIC) [19] and as well as the DICr [20], which is more appropriate for the mixture model, were used for model evaluation in this study. An exceedance probability was used to examine localized behaviour of a model; this is one of the main tools for determining unusual elevations of disease. The exceedance probability is usually calculated from the posterior sample values and is defined as  where G is the sampler sample size. There are two components in the exceedance probability to be assessed. The first one is the cutoff point *c* for the theta, and it can be 1, 2 or 3, for the extent of extreme risk. The second part is an exceedance probability threshold to an unusual risk area. Some of the selected thresholds are 0.95, 0.975, and 0.99 for P (θ_i_ > c). The levels of extreme risk depend on the values of *c* [21].

## Results

### Temporal pattern of malaria cases

**Fig. 2:**
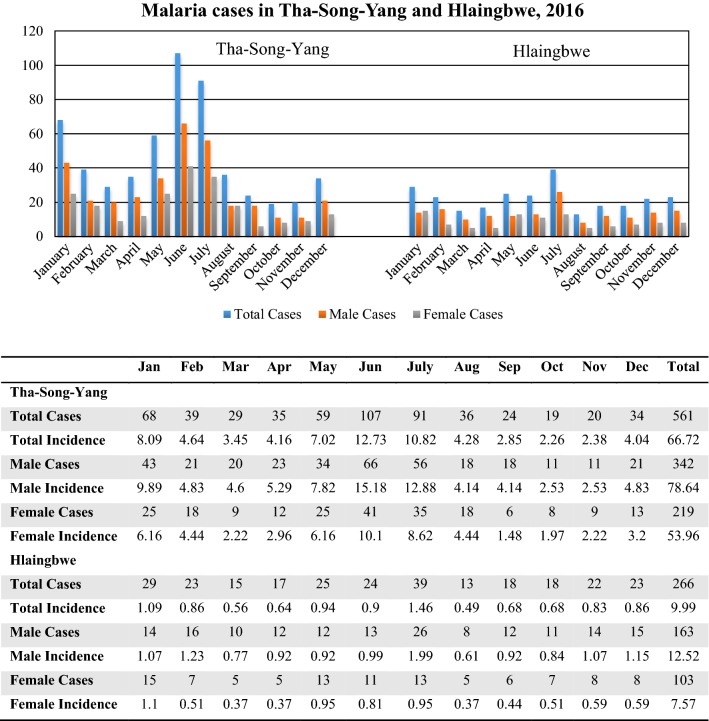
Malaria cases and incidences in Tha-Song-Yang and Hlaingbwe, 2016

In 2016, there were a total 266 cases (incidence: 9.99 per 10,000) in Hlaingbwe Township and 561 cases (incidence: 66.72 per 10,000) in Tha-Song-Yang District. Similar patterns observed in both Hlaingbwe Township and Tha-Song-Yang District revealed a seasonal pattern with two major peaks, during May–June–July and December–January–February. As shown in Fig. 2, the highest number of malaria cases in the border township-district was found during the rainy season from May to July, and another high number of cases was seen in January, in comparison with other months. When classified by gender, the overall ratio of male:female cases in Hlaingbwe Township was 163:103 (incidence per 10,000, ratio: 12.52:7.57); in Tha-Song-Yang, it was 342:219 (incidence ratio: 78.64:53.96). This demonstrated that approximately two-thirds of the total cases involved males in both Tha-Song-Yang and Hlaingbwe. The pattern of malaria cases reported on a monthly basis was similar for both gender, as shown in Fig. 2.

**Fig. 3:**
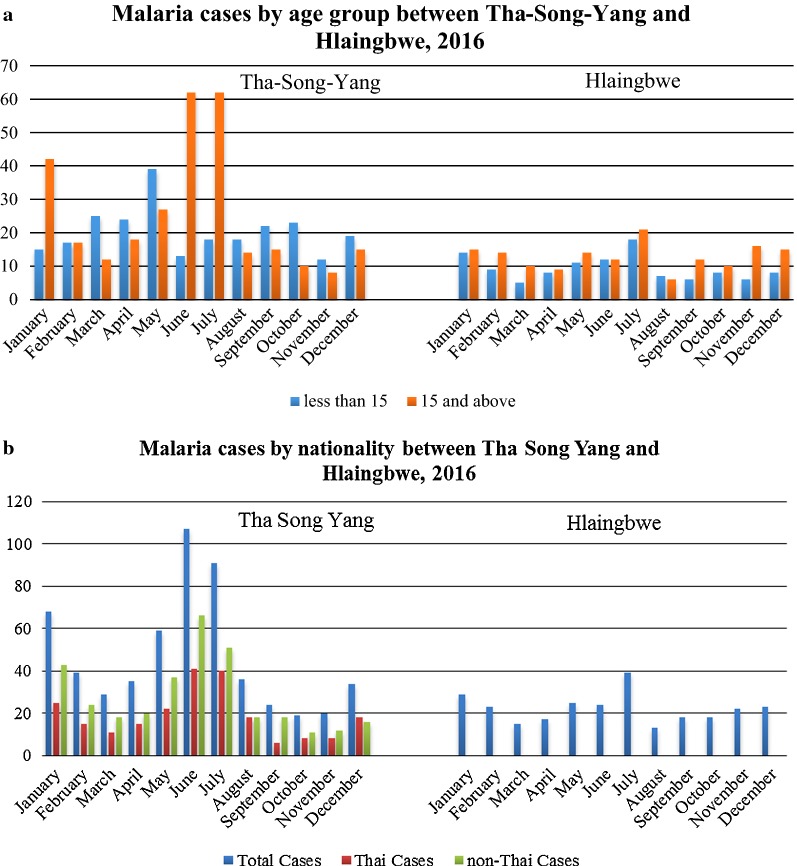
Malaria cases in Tha-Song-Yang and Hlaingbwe in 2016, classified by different demographic characteristics

When classified by age group, the ratio of malaria cases involving individuals aged ≥ 15 years versus < 15 years in Hlaingbwe Township was 154:112 and that in Tha-Song-Yang was 302:259. The result showed that among male cases, there were more cases among adults than younger males; 65.64% vs. 34.36% for Hlaingbwe Township, and 58.77% vs. 41.23% in Tha-Song-Yang District (Table 1). Contradictorily, among female cases, there was more cases among younger females than adult females; 54.37% vs. 45.63% in Hlaingbwe Township and 53.88% vs. 46.12% in Tha-Song-Yang District. Interestingly, more malaria infections occurred in the age group of ≥ 15 years in Hlaingbwe Township. It should be noted, however, that the ratios of malaria cases by age group in Tha-Song-Yang District were not consistent across the 12 months; there were more cases among individuals aged ≥ 15 years during the peak months (January, June, July) but more cases among those aged < 15 years in other months of the year, as shown in Fig. 3a. For malaria cases in Tha-Song-Yang District, there were more non-Thai people with malaria than cases among Thais across all months of the year; the cases in Hlaingbwe Township were all reported to be Myanmar nationals, as shown in Fig. 3b.

**Table 1:** Malaria cases classified by gender and age groups in Tha-Song-Yang and Hlaingbwe, 2016

Gender/age	Tha-Song-Yang			Hlaingbwe Township		
	< 15 years	≥ 15 years	Total	< 15	≥ 15	Total
Male	141 (41.23%)	201 (58.77%)	342	56 (34.36%)	107 (65.64%)	163
Female	118 (53.88%)	101 (46.12%)	219	56 (54.37%)	47 (45.63%)	103
Total	259	302	561	112	154	266

### Spatial distribution of malaria cases and incidences

**Fig. 4:**
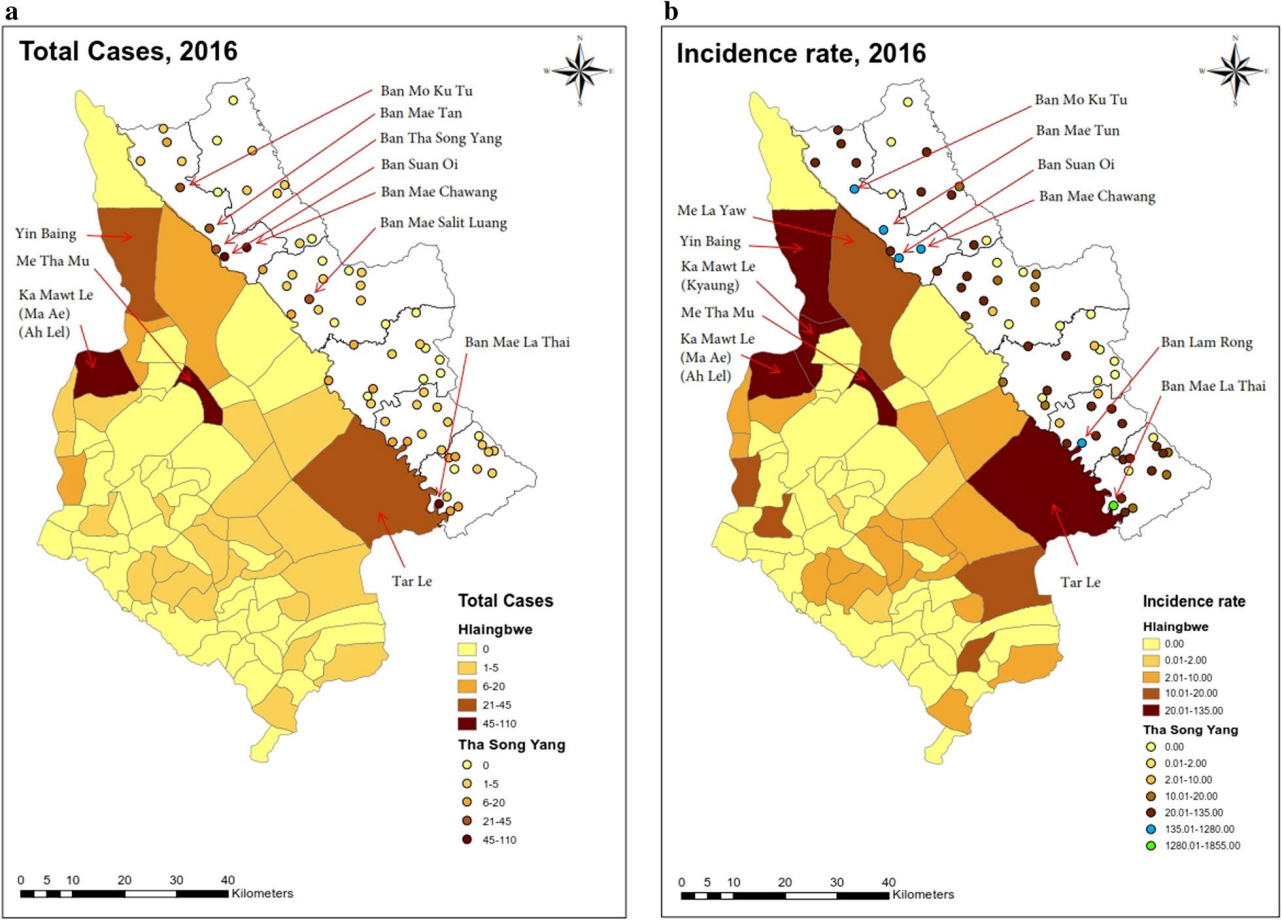
Total malaria cases and incidences in Tha-Song-Yang and Hlaingbwe regions during 2016.** a** Total malaria cases.** b** Incidence rate per 10,000 population

As shown in Fig. 4, mapping of malaria cases and incidences in Hlaingbwe Township was presented using village-tract shapes within the township; those in Tha-Song-Yang District were plotted using village centre points (as there were no shape files for the villages). Mapping of the total cases over the year (Fig. 4a) revealed that cases were scattered over more than half of the area (50 of 66 villages) in Tha-Song-Yang District and over approximately half of the area (30 of 75 village tracts) in Hlaingbwe Township. Mapping of the incidences over the year (Fig. 4b) showed that villages and village tracks with high numbers of cases were most likely to be those with high incidence rates per 10,000 population size. A higher number of malaria cases and incidences could be seen in the inner and northern of Hlaingbwe Township, except Tar Le, which is situated along the Thai–Myanmar border. The village tracts in the inner and upper side of Hlaingbwe Township with a high number of cases (> 20 cases) included: Yin Baing, Me Tha Mu, Ka Mawt Le (Ma Ae) (Ah Lel) and Tar Le; those with high incidence rates (> 10 per 10,000 population) also included Yin Baing, Me Tha Mu, Ka Mawt Le (Ma Ae) (Ah Lel), Tar le, Ka Mawt Le (Kyaung) and Me LaYaw. In the Tha-Song-Yang region, a higher number of malaria cases (> 20 cases) occurred in Ban Mo Ku Tu, Ban Mae Tun, Ban Tha-Song-Yang, Ban Suan Oi, Ban Mae Chawang, Ban Mae Salit Luang, and Ban Mae La Thai. The incidence rates were much higher in Tha-Song-Yang District compared to those in Hlaingbwe Township. High incidence rates (> 10 per 10,000 population) were shown in about half of the total number of villages; similar to villages with high number of cases, those with very high incidence rate (> 135 per 10,000 population) included Ban Mo Ku Tu, Ban Mae Tun, Ban Suan Oi, Ban Mae Chawang, Ban Lam Rong, and Ban Mae La Thai All of these are situated in both the inner side and along the Thai–Myanmar border (Fig. 4).

### Bayesian spatiotemporal models

**Fig. 5:**
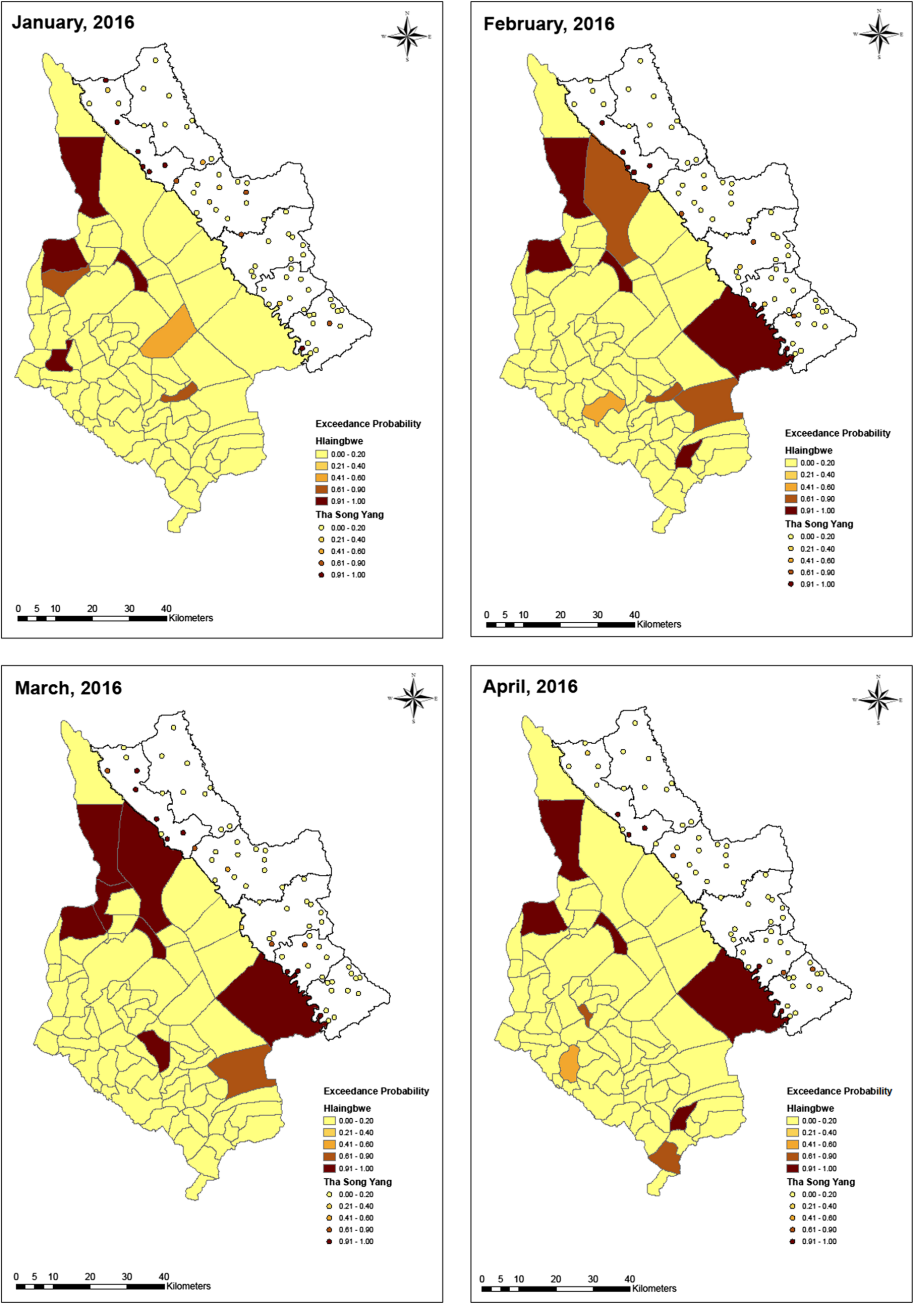
Exceedance probability of relative risk in Tha-Song-Yang and Hlaingbwe regions from January to April, 2016

**Fig. 6:**
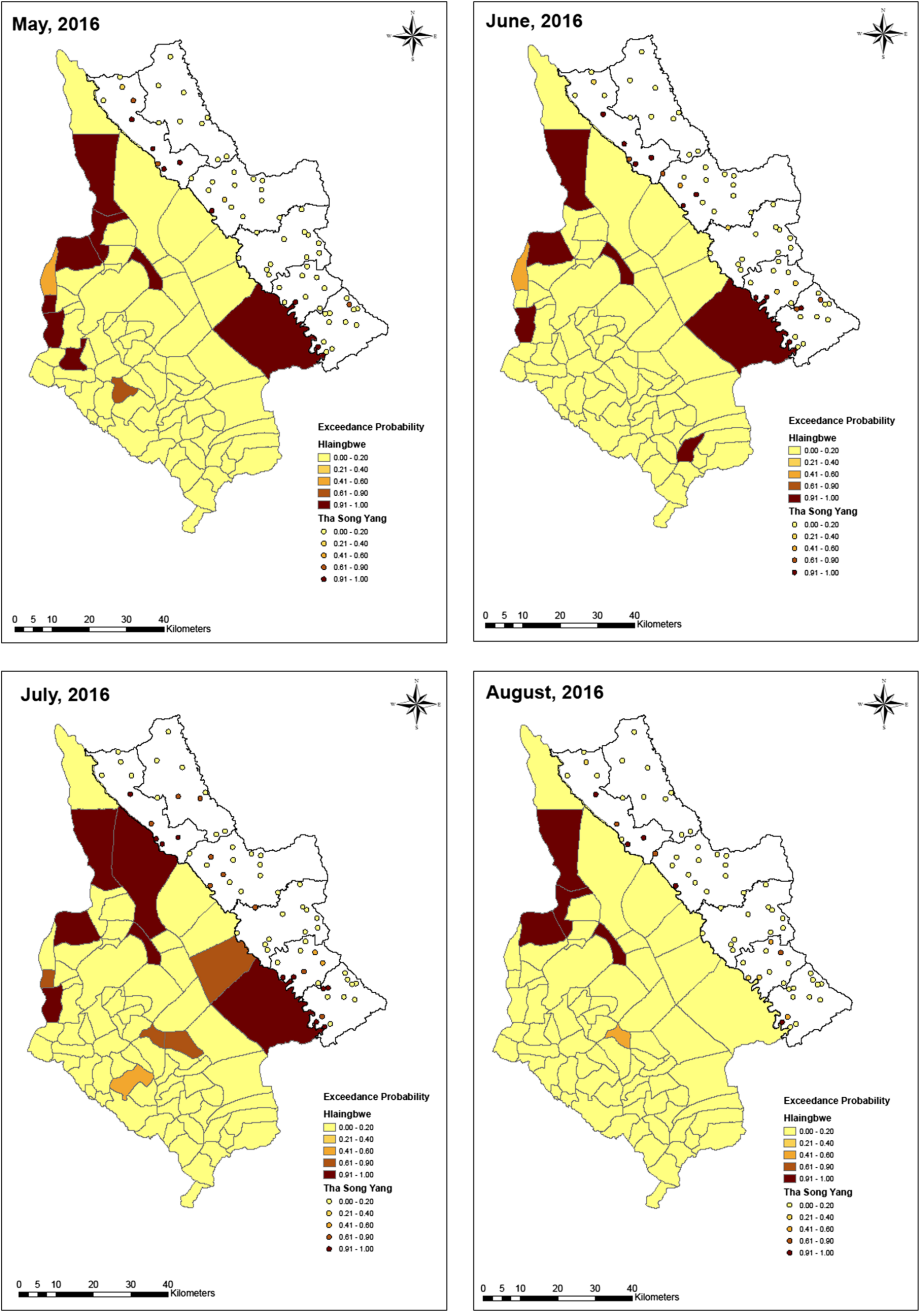
Exceedance probability of relative risk in Tha-Song-Yang and Hlaingbwe regions from May to August, 2016

**Fig. 7:**
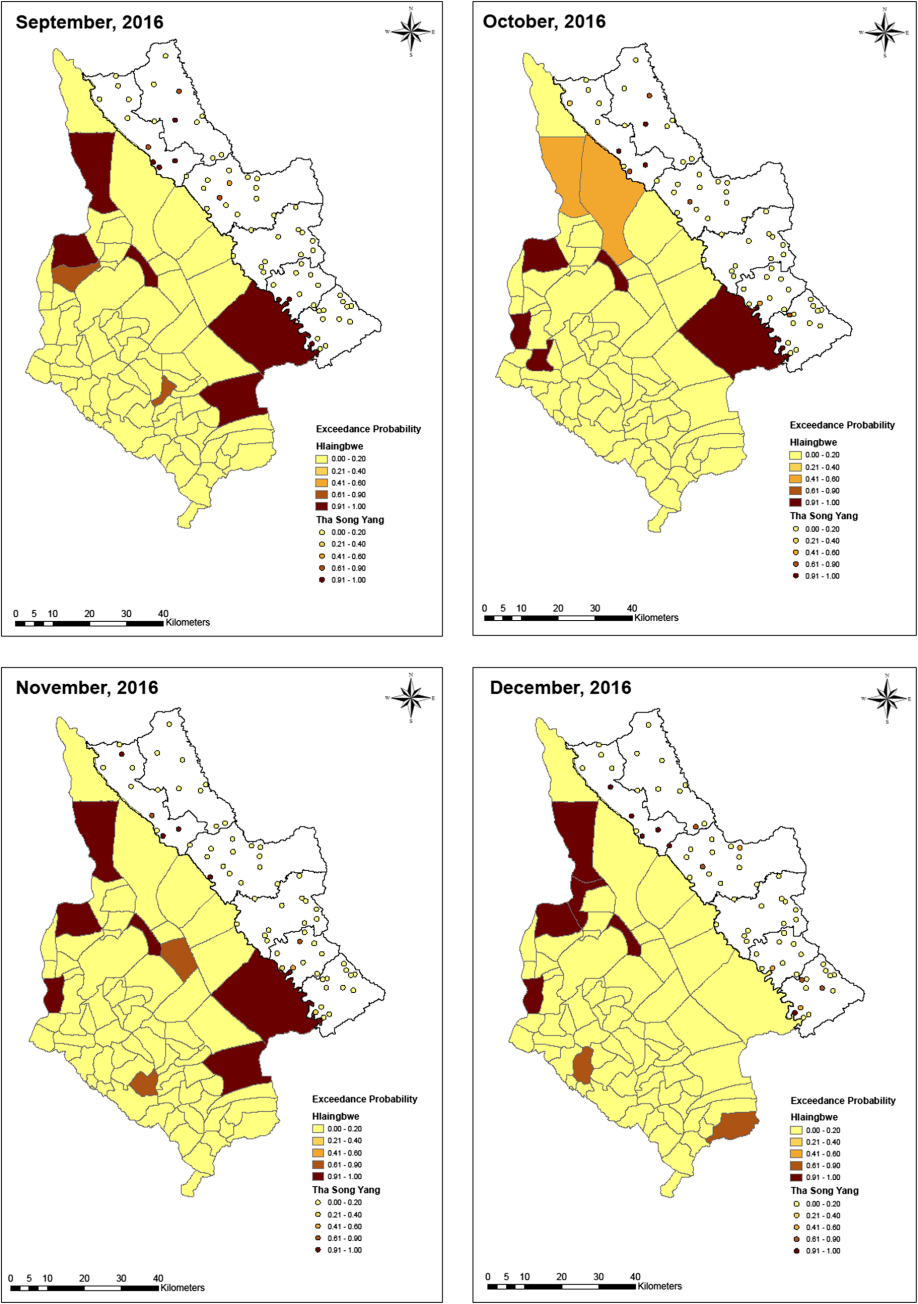
Exceedance probability of relative risk in Tha-Song-Yang and Hlaingbwe regions from September to December, 2016

Figures 4, 5, 6 and 7 show several study areas had no cases in months during 2016 which indicates a potential issue of excess zeros. The zero-inflated Poisson (ZIP) regression model then was fitted to link the count data of monthly malaria cases in different villages (Tha-Song-Yang) and village tracts (Hlaingbwe) with 3 observed variables (covariates) including gender (male *vs* female cases), age group (≥ 15 years vs < 15 years), and interaction between age and gender. Table 2 describes the results for regression spatiotemporal coefficients of the covariates as predictors of monthly malaria cases in Tha-Song-Yang District and Hlaingbwe Township. The 3 covariates were statistically significant in the model. In the Tha-Song-Yang and Hlaingbwe models, the incidence rate ratios of malaria infection among those aged ≥ 15 years compared with those aged < 15 years were 1.87 (95% CI 1.31, 2.48) and 2.82 (95% CI 2.03, 3.62), respectively. Regarding gender, in the Tha-Song-Yang and Hlaingbwe models, the incidence rate ratios of malaria infection among male compared with female cases were 2.27 (95% CI 1.71, 2.86) and 2.87 (95% CI 2.07, 3.68), respectively. The interaction between age and gender showed negative associations with malaria cases: for the Tha-Song-Yang model, the incidence rate ratio of malaria infection was − 2.68 (95% CI − 3.50, − 1.88) and for the Hlaingbwe model was − 3.45 (95% CI − 4.58, − 2.35).

**Table 2:** Regression coefficients with 95% credible interval and deviance information criterion (DIC) from zero-inflated Poisson models for malaria cases in 2016

Model/variables	Tha-Song-Yang model	Hlaingbwe model
Zero-inflated Poisson		
Intercept	− 1.38 (− 1.90, − 0.84)	− 1.99 (− 2.65, − 1.37)
Age (> 15 years)	1.87 (1.31, 2.48)	2.82 (2.03, 3.62)
Sex (male)	2.27 (1.71, 2.86)	2.87 (2.07, 3.68)
Age and sex	− 2.68 (− 3.50, − 1.88)	− 3.45 (− 4.58, − 2.35)
DIC	935.51	477.58
DICr	939.53	479.22

### Exceedance probability map

Based on the ZIP model, the exceedance probability of relative risk was estimated with a cut-off point of 1. As shown in Figs. 5, 6 and 7, maps of hotspot clusters were generated for each month over the year. For Tha-Song-Yang District, the disease-clustering areas were indicated in villages in the upper (Tha-Song-Yang sub-district), middle (Mae Song and Mae U Su sub-districts), and lower (Mae Tan and Mae La sub-districts) parts of the area along the Thai–Myanmar border, particularly during the highest malaria infection months (June and July), and only in the upper and lower parts for the other months. Some apparent hotspots appeared along the border and some were scattered over areas including Mae Wa Luang sub-district, which is far from the border area in Tha-Song-Yang District. In Hlaingbwe Township, most of the aggregated risk occurred in village tracts farther from the border, and frequently occurred in the northern part. However, it can be seen that some aggregated risk with an excess of higher thresholds in this region existed in areas adjacent to Tha-Song-Yang District during some months. Some apparent hotspots could be seen spread out over interior parts of Hlaingbwe, far from the border area, and only one or two hotspots were situated along the Thai–Myanmar border during some months.

## Discussion

This study used data from malaria surveillance systems collected by the healthcare sectors in the adjacent township-district of Myanmar and Thailand, where malaria cases have been consistently reported. In addition to basic spatial and temporal analyses, Bayesian spatiotemporal ZIP model was developed to determine the probability of aggregated risk in each village tract or village of the two study areas.

As reported in many other studies [22–24], there are two peaks of malaria infection in this region, with the higher peak during the rainy months when there is abundant rainfall. Such incidence can be explained by proliferation of the vector in aquatic habitats and more work-related activities in the agricultural sector than in other sectors in both study areas. It is clear that there were many more cases in Tha-Song-Yang District than in the adjacent Hlaingbwe Township. However, from the data shown in this study, more than half of the monthly reported malaria cases in Tha-Song-Yang District involved non-Thai individuals. Migration, as well as work-related population movement in this region, has been indicated as important factors contributing to malaria epidemiology [25–35]. Moreover, displaced minority populations also increase the risk of malaria infection in this region; it has been suggested in the literature that political instability among minority ethnic groups in Myanmar has led to considerable cross-border population movement. Among non-Thais, migrant workers from Myanmar represent the largest population of foreign workers [25, 36]. There are also displaced people without a nationality and illegal immigrants in considerable numbers [37, 38]. A study of mosquito vectors in these study areas found that *P. falciparum* infections were more concentrated seasonally among the recent migrant population while *P. vivax* cases were significantly associated with the dynamics of the local mosquito population and less with migrant status [37]. It would be interesting for further study to collect the detailed migration patterns of the infected cases prospectively, rather than using surveillance data, and then performing model fit with variations in risk based on species together with other specific host characteristics.

Some previous studies in the Greater Mekong Sub-region have indicated that malaria clustered along the international borders is associated with forests and forest edges [2, 25, 39]. Even though in the present study, there was no analysis of the association of the number of malaria cases with geographical features, it can be seen from the map of malaria case/incidence distribution that the border areas with high mountains and heavily forested terrain, in some upper and middle areas along the border, tended to have fewer malaria cases. On the other hand, there appeared to be more cases/incidences in village tracts or villages along the border that are situated on a flat plain or with a shallow river as a natural demarcation line. Particularly along the lower plain agricultural zone, large mobile cross-border population movement could occur in both directions between the two countries. With year-round movement as well as river crossings, migrants from a malaria-endemic zone can bring the parasite to a new, non-malaria zone [40]. The cross-border workforce that spends time on either side in the malaria-endemic area can be infected with the parasite and may then carry it back and forth across the border [2]. Studies on the spread of drug-resistant strains [41–44] have noted that migrants can transport drug-resistant strains from visited areas to new locations, regardless of whether malaria is present or not in these new sites. This means that individuals who enter malaria regions can influence epidemiological dynamics [45, 46]. This situation along the border represents a difficult challenge for the management of imported malaria on both sides.

Similar to other studies regarding the epidemiological dynamics of risk factors for malaria infection [3, 23, 47], there were more malaria cases among males in both study sites. In both Tha-Song-Yang District and Hlaingbwe Township, the number of cases and the incidence of malaria infection among males appear to be higher than females throughout the year. The observation of differences in infection rates by gender may indicate behavioural differences and occupational orientations. The main reason is clearly related to the nature of men’s work, in high-risk areas with high human-vector contact such as on farms, in orchards, or in forests. However, it should be noted that the number of cases of malaria among women was not significantly lower than that among men. The target groups should include both gender in planning strategies for malaria prevention and control. Regarding malaria infection among different age groups in the study areas, it is interesting to see that there were more cases among those aged ≥ 15 years in Hlaingbwe Township, but more cases among those aged < 15 years in Tha-Song-Yang District. It should be noted, however, that this observation is based on case counts and not incidence (as there were no denominators for age groups). Malaria infection in the higher age group (≥ 15 years) might be associated with individuals who engaged in more activities in high-risk areas and used inadequate protective measures; it could also be related to lower natural immunity with increased age [48]. A cross-sectional survey of sub-clinical malaria infections in Southeast Asia, including similar Thai–Myanmar border areas, also reported the age distributions of their study participants; the median age was 21 years with 37% under 15 years of age [49]. Another report of malaria case findings among mobile populations and migrant workers in Myanmar indicated that migrant workers from rural areas were likely to migrate to other rural areas and the majority of migrants were men (> 60%) with about 80% between the ages of 11 and 30 years [50]. However, the higher number of malaria infections among those aged < 15 years in Tha-Song-Yang District might be owing to the fact that the children often accompany their parents to their workplaces or to forests where vectors thrive, or they spend their time in or near the home or at school in areas with high vector populations [51]. A study on the ecology and epidemiology of malaria along the Thai–Myanmar border suggested that the presence and distribution of mosquito vectors was directly related to the availability of hosts and contact patterns between vectors and hosts; the biting habits of the mosquito vectors abundant in the region occurred as frequently indoors as outdoors in open houses in forests [52]. It was also noted that their feeding patterns (early versus late) were sometimes contradictory, even in the same site and species across different years or locations. A few studies of the entomological determinants of malaria transmission in the same areas as this study along the Thai–Myanmar border also reported that *Anopheles* mosquitoes exhibited an outdoor and early biting pattern with active timing between 06:00 and 07:00 [53, 54]. Several studies have reported high malaria morbidity among children [55, 56]; interestingly, one study in the Laiza refugee camp along the Myanmar–China border [57] speculated that daytime malaria transmission might occur near the primary school attended by younger children. It would be interesting to conduct an entomological investigation to explore this hypothesis in Tha-Song-Yang District. However, there is a possibility that this high morbidity is related to unequal health service utilization or variation in behavioural exposure to disease.

Regarding the ZIP model, all of the covariates including age, gender and interaction of age and gender showed statistically significant associations with the number of malaria cases. This confirmed that, in general, malaria cases occur more often among males and those aged ≥ 15 years in the study area. It is interesting that the interaction of the covariates age and gender had a negative relationship with malaria infection cases. The interaction could be explained as shown in Table 1 such that there were more cases among adult males than younger males in contrast to more cases among younger females than adult females. Similar to other studies [49, 58], there were differences between children and adults for malaria cases among both females and males. This finding of more infections among adult males was consistent with those of other studies [33, 59, 60], which might be associated with their work-related activities in high-risk areas previously discussed. However, with respect to the high number of malaria cases among younger females, it could be that some transmission was occurring in or close to schools or areas where children spend most of their time, as previously discussed [51], or it could be speculated that when girls become adults, they tend to go outside less and are more likely to work indoors or in less risky settings. The contradicting number of malaria cases among different age groups and gender in both Tha-Song-Yang District and Hlaingbwe Township requires further investigation.

Based on the ZIP model, the exceedance probabilities of relative risk with a threshold of 0.9 was developed to detect disease clustering by vigorously put forward localized concentrations, which are different from isolated hotspots [21]. Mapping to detect hotspot clusters using exceedance probability in this study revealed that hotspots in risky areas could vary across spatial and temporal parameters. Some village tracts/villages had a consistently high probability of malaria infection whereas others had consistently moderate or low probability of malaria infection. The probability of becoming a hotspot, however, varied among certain village tracts/villages in different months, with some having nearly no cases at all times. It is also interesting to note that the probabilities of hotspot development varied over the year in the township-district adjacent to the border. This model can be helpful in characterizing the spatiotemporal pattern of malaria and deciding linkages between spatiotemporal patterns and driving factors of malaria transmission risk. Appropriate intervention and resource allocation can then be managed in respective areas if the government as well as malaria control and prevention partners have better knowledge of the spatiotemporal clustering of malaria.

## Limitations of the study

The models developed in this study were based on only the association between human population density and malaria cases in space and time, with the parameters age and gender. There might be several other important factors that affect malaria incidence that could be applied, to obtain a better model. It should be noted that the data used in this study were secondary data from government surveillance systems. These data may have limitations in terms of data quality such as completeness, underreported data and validity. Some data were unavailable (e.g., number of non-Thai or migrant workers, and age groups at township or district levels); the analyses had to be based on the number of cases rather than the incidence.

## Conclusions

In this study, a Bayesian spatial and temporal model was performed to assess malaria infections along the township-district adjacent to the Myanmar–Thailand border. The findings of this study confirmed commonly known information about risk factors regarding gender and age groups for cases of malaria infection. However, contradicting proportions of malaria cases among the different gender and age groups were noted in this study. The analytic model developed in this study could be used to assess the probability of hotspot areas, which could be beneficial for establishing priority zones in preventive actions, with appropriate timetables in high-risk border areas. To obtain a more inclusive view of malaria risk, a future advanced model is planned, to include infectivity, densities and distribution of the vector, identifiable breeding sites of the vector, as well as climatic and environmental factors, as the underlying causes of increased risk in the identified areas.

## Additional files

**Additional file 1: Table S1.** Name of villages, Tha-Song-Yang District.

**Additional file 2: Table S2.** Name of village tracts, Hlaingbwe Township.
