# Supplementary material for: Bayesian spatiotemporal analysis of malaria infection along an international border: Hlaingbwe Township in Myanmar and Tha-Song-Yang District in Thailand
Source: Malar J. 2018 Nov 16;17:428. doi: 10.1186/s12936-018-2574-0 (PMC6240260; doi:10.1186/s12936-018-2574-0)
Supplement: Supplementary file 1 — Additional file 1: Table S1. Name of villages, Tha-Song-Yang District. [file 12936_2018_2574_MOESM1_ESM.docx]

Table S1. Name of villages, Tha-Song-Yang district

| Province Code | District Code | Subdistrict Code | Village Code | Village Name |
| --- | --- | --- | --- | --- |
| 63 | 6305 | 630501 | 63050101 | Ban Tha Song Yang |
| 63 | 6305 | 630501 | 63050102 | Ban Mae Tun |
| 63 | 6305 | 630501 | 63050103 | Ban Mo Ku Tu |
| 63 | 6305 | 630501 | 63050104 | Ban Mae Woei |
| 63 | 6305 | 630501 | 63050105 | Ban Khun Mae Woei |
| 63 | 6305 | 630501 | 63050106 | Ban Mae Lana |
| 63 | 6305 | 630501 | 63050107 | Ban Mae Om Ya |
| 63 | 6305 | 630501 | 63050108 | Ban Mae Chawang |
| 63 | 6305 | 630501 | 63050109 | Ban Suan Oi |
| 63 | 6305 | 630502 | 63050201 | Ban Mae Tan |
| 63 | 6305 | 630502 | 63050202 | Ban Lam Rong |
| 63 | 6305 | 630502 | 63050203 | Ban Khun Huai Mae Khamo |
| 63 | 6305 | 630502 | 63050204 | Ban Bae Ro Tha |
| 63 | 6305 | 630502 | 63050205 | Ban Huai Pu Kaeng |
| 63 | 6305 | 630502 | 63050206 | Ban Khun Huai Mae Tan |
| 63 | 6305 | 630502 | 63050207 | Ban Mae Pho |
| 63 | 6305 | 630502 | 63050208 | Ban Huai Mae Khamo |
| 63 | 6305 | 630502 | 63050209 | Ban Thung Tham |
| 63 | 6305 | 630502 | 63050210 | Ban Thung Na Sung |
| 63 | 6305 | 630503 | 63050301 | Ban Mae Song |
| 63 | 6305 | 630503 | 63050302 | Ban Mae Salit Luang |
| 63 | 6305 | 630503 | 63050303 | Ban Mae Salit Noi |
| 63 | 6305 | 630503 | 63050304 | Ban Re Wo Kro |
| 63 | 6305 | 630503 | 63050305 | Ban Mae Kho |
| 63 | 6305 | 630503 | 63050306 | Ban Mae Nin |
| 63 | 6305 | 630503 | 63050307 | Ban Huai Manok |
| 63 | 6305 | 630503 | 63050308 | Ban Mae Ramoeng |
| 63 | 6305 | 630503 | 63050309 | Ban Ta Phi Doe |
| 63 | 6305 | 630503 | 63050310 | Ban Khloe De Khi |
| 63 | 6305 | 630503 | 63050311 | Ban Bo Bo Khi |
| 63 | 6305 | 630503 | 63050312 | Ban Wa Do Kro |
| 63 | 6305 | 630503 | 63050313 | Ban Thi Bo Khi |
| 63 | 6305 | 630503 | 63050314 | Ban King Mai Khao |
| 63 | 6305 | 630503 | 63050315 | Ban Mae Pha Daeng |
| 63 | 6305 | 630503 | 63050316 | Ban San Doi Ngam |
| 63 | 6305 | 630504 | 63050401 | Ban Mae La Thai |
| 63 | 6305 | 630504 | 63050402 | Ban Mae La Yang |
| 63 | 6305 | 630504 | 63050403 | Ban Mae Ok Pha Ru |
| 63 | 6305 | 630504 | 63050404 | Ban Huai Nok Kok |
| 63 | 6305 | 630504 | 63050405 | Ban Ka Ma Pha Do |
| 63 | 6305 | 630504 | 63050406 | Ban Khun Huai Mae La |
| 63 | 6305 | 630504 | 63050407 | Ban Khun Huai Nok Kok |
| 63 | 6305 | 630504 | 63050408 | Ban Thi Chu Lo Khi |
| 63 | 6305 | 630504 | 63050409 | Ban Mae Ok Hu |
| 63 | 6305 | 630504 | 63050410 | Ban Pha No Di |
| 63 | 6305 | 630504 | 63050411 | Ban Mae La Pho Di |
| 63 | 6305 | 630504 | 63050412 | Ban Mae La Khi |
| 63 | 6305 | 630505 | 63050501 | Ban So Khae Ra Ka |
| 63 | 6305 | 630505 | 63050502 | Ban Pang Thong |
| 63 | 6305 | 630505 | 63050503 | Ban Mae Wa Luang |
| 63 | 6305 | 630505 | 63050504 | Ban Mae Om Ki |
| 63 | 6305 | 630505 | 63050505 | Ban Bo Ma |
| 63 | 6305 | 630505 | 63050506 | Ban Mae La Yo Khi |
| 63 | 6305 | 630505 | 63050507 | Ban Om Ko Tha |
| 63 | 6305 | 630505 | 63050508 | Ban Wa Ya Cho |
| 63 | 6305 | 630505 | 63050509 | Ban Kre Ko |
| 63 | 6305 | 630506 | 63050601 | Ban Khane Chu |
| 63 | 6305 | 630506 | 63050602 | Ban Mo Ko Di |
| 63 | 6305 | 630506 | 63050603 | Ban Mae U Su |
| 63 | 6305 | 630506 | 63050604 | Ban Thi No Kho |
| 63 | 6305 | 630506 | 63050605 | Ban Re Kati |
| 63 | 6305 | 630506 | 63050606 | Ban Nong Bua |
| 63 | 6305 | 630506 | 63050607 | Ban Ta Ko Khi |
| 63 | 6305 | 630506 | 63050608 | Ban Mae Song Noi |
| 63 | 6305 | 630506 | 63050609 | Ban Mo Thi Tha |
| 63 | 6305 | 630506 | 63050610 | Ban Dok Mai Sot |
