# Supplementary material for: Bayesian spatiotemporal analysis of malaria infection along an international border: Hlaingbwe Township in Myanmar and Tha-Song-Yang District in Thailand
Source: Malar J. 2018 Nov 16;17:428. doi: 10.1186/s12936-018-2574-0 (PMC6240260; doi:10.1186/s12936-018-2574-0)
Supplement: Supplementary file 2 — Additional file 2: Table S2. Name of village tracts, Hlaingbwe Township. [file 12936_2018_2574_MOESM2_ESM.docx]

Table S2 Name of Village Tracts, Hlaingbwe Township

| State Pcode | District Pcode | Township Pcode | Village tract Pcode | Village tract |
| --- | --- | --- | --- | --- |
| MMR003 | MMR003D001 | MMR003002 | MMR003002024 | Kyar Inn |
| MMR003 | MMR003D001 | MMR003002 | MMR003002040 | Thar Yar Kone (Kat Pa Li) |
| MMR003 | MMR003D001 | MMR003002 | MMR003002004 | Naung Taing |
| MMR003 | MMR003D001 | MMR003002 | MMR003002020 | Nwet Pyin Nyar |
| MMR003 | MMR003D001 | MMR003002 | MMR003002003 | Win Sein |
| MMR003 | MMR003D001 | MMR003002 | MMR003002005 | Kun Bi |
| MMR003 | MMR003D001 | MMR003002 | MMR003002021 | Me Law Khee |
| MMR003 | MMR003D001 | MMR003002 | MMR003002071 | Pa Hta Lar Pa (Paingkyon Sub-township) |
| MMR003 | MMR003D001 | MMR003002 | MMR003002069 | Tha Mo (Paingkyon Sub-township) |
| MMR003 | MMR003D001 | MMR003002 | MMR003002068 | Mi Kyaung (Paingkyon Sub-township) |
| MMR003 | MMR003D001 | MMR003002 | MMR003002036 | Kyon Pa Ko |
| MMR003 | MMR003D001 | MMR003002 | MMR003002037 | Kyoet Chaung |
| MMR003 | MMR003D001 | MMR003002 | MMR003002039 | Ta Khin Lone |
| MMR003 | MMR003D001 | MMR003002 | MMR003002015 | Tha Pu To |
| MMR003 | MMR003D001 | MMR003002 | MMR003002014 | Naung Tha Nge |
| MMR003 | MMR003D001 | MMR003002 | MMR003002013 | Than Ban |
| MMR003 | MMR003D001 | MMR003002 | MMR003002017 | Ka Ti Kan |
| MMR003 | MMR003D001 | MMR003002 | MMR003002012 | Hti Lon |
| MMR003 | MMR003D001 | MMR003002 | MMR003002011 | Auk Yae Pu |
| MMR003 | MMR003D001 | MMR003002 | MMR003002010 | Ah Htet Yae Pu |
| MMR003 | MMR003D001 | MMR003002 | MMR003002000 | Hti Lon R.F |
| MMR003 | MMR003D001 | MMR003002 | MMR003002008 | Daing Pya |
| MMR003 | MMR003D001 | MMR003002 | MMR003002002 | Kawt Hlaing |
| MMR003 | MMR003D001 | MMR003002 | MMR003002044 | Ka So (Paingkyon Sub-township) |
| MMR003 | MMR003D001 | MMR003002 | MMR003002046 | Moe Naing (Paingkyon Sub-township) |
| MMR003 | MMR003D001 | MMR003002 | MMR003002048 | Naung Boe Gy (Paingkyon Sub-township)i |
| MMR003 | MMR003D001 | MMR003002 | MMR003002049 | Ywar Kaing Kaung (Paingkyon Sub-township) |
| MMR003 | MMR003D001 | MMR003002 | MMR003002062 | Paw Lauk (Paingkyon Sub-township) |
| MMR003 | MMR003D001 | MMR003002 | MMR003002063 | Htan Ta Pin (Paingkyon Sub-township) |
| MMR003 | MMR003D001 | MMR003002 | MMR003002061 | Kyet Tu Yway (Paingkyon Sub-township) |
| MMR003 | MMR003D001 | MMR003002 | MMR003002059 | Hti Hpoe Kein (Paingkyon Sub-township) |
| MMR003 | MMR003D001 | MMR003002 | MMR003002070 | Paung (Paingkyon Sub-township) |
| MMR003 | MMR003D001 | MMR003002 | MMR003002057 | Bi Sa Kat (Paingkyon Sub-township) |
| MMR003 | MMR003D001 | MMR003002 | MMR003002051 | Naung Mya Lwe (Paingkyon Sub-township) |
| MMR003 | MMR003D001 | MMR003002 | MMR003002055 | Pa Ta (Paingkyon Sub-township) |
| MMR003 | MMR003D001 | MMR003002 | MMR003002060 | Yae Pu Gyi (Paingkyon Sub-township) |
| MMR003 | MMR003D001 | MMR003002 | MMR003002056 | Naung Khwee (Paingkyon Sub-township) |
| MMR003 | MMR003D001 | MMR003002 | MMR003002058 | Tar Ka Yar (Paingkyon Sub-township) |
| MMR003 | MMR003D001 | MMR003002 | MMR003002052 | Kawt Pa Mu (Paingkyon Sub-township)t |
| MMR003 | MMR003D001 | MMR003002 | MMR003002018 | Kawt Myat Gyi |
| MMR003 | MMR003D001 | MMR003002 | MMR003002038 | U Daung |
| MMR003 | MMR003D001 | MMR003002 | MMR003002030 | Ka Mawt Le (Ma Ae) (Ah Lel) |
| MMR003 | MMR003D001 | MMR003002 | MMR003002033 | Mya Lay |
| MMR003 | MMR003D001 | MMR003002 | MMR003002034 | Inn No Theik Pan |
| MMR003 | MMR003D001 | MMR003002 | MMR003002031 | Shwe Gun |
| MMR003 | MMR003D001 | MMR003002 | MMR003002064 | Me Pa Ra (Paingkyon Sub-township) |
| MMR003 | MMR003D001 | MMR003002 | MMR003002042 | Ta Khwet Hpoe |
| MMR003 | MMR003D001 | MMR003002 | MMR003002006 | Pein Hne Taw |
| MMR003 | MMR003D001 | MMR003002 | MMR003002701 | Urban |
| MMR003 | MMR003D001 | MMR003002 | MMR003002007 | Kun Tar |
| MMR003 | MMR003D001 | MMR003002 | MMR003002001 | Ka Mawt Ka Chu |
| MMR003 | MMR003D001 | MMR003002 | MMR003002050 | Kawt Nwet (Paingkyon Sub-township) |
| MMR003 | MMR003D001 | MMR003002 | MMR003002047 | Win Pa Ya (Paingkyon Sub-township) |
| MMR003 | MMR003D001 | MMR003002 | MMR003002016 | Tar Paung |
| MMR003 | MMR003D001 | MMR003002 | MMR003002053 | Kawt Ma Yaing (Paingkyon Sub-township) |
| MMR003 | MMR003D001 | MMR003002 | MMR003002066 | Win Saw (Paingkyon Sub-township) |
| MMR003 | MMR003D001 | MMR003002 | MMR003002043 | Hta Thein Kyoe Waing |
| MMR003 | MMR003D001 | MMR003002 | MMR003002023 | Kwee Lay |
| MMR003 | MMR003D001 | MMR003002 | MMR003002000 | Hlaingbwe R.F |
| MMR003 | MMR003D001 | MMR003002 | MMR003002022 | Me Tha Mu |
| MMR003 | MMR003D001 | MMR003002 | MMR003002027 | Yae Ta Khun |
| MMR003 | MMR003D001 | MMR003002 | MMR003002026 | Yin Baing |
| MMR003 | MMR003D001 | MMR003002 | MMR003002072 | Poe Pa Lay (Paingkyon Sub-township) |
| MMR003 | MMR003D001 | MMR003002 | MMR003002029 | Me La Yaw |
| MMR003 | MMR003D001 | MMR003002 | MMR003002025 | Saw Law |
| MMR003 | MMR003D001 | MMR003002 | MMR003002065 | Tar0 Le (Paingkyon Sub-township)l |
| MMR003 | MMR003D001 | MMR003002 | MMR003002067 | Law Kaw (Paingkyon Sub-township) |
| MMR003 | MMR003D001 | MMR003002 | MMR003002009 | Sin Ku |
| MMR003 | MMR003D001 | MMR003002 | MMR003002019 | Pat Kyaw |
| MMR003 | MMR003D001 | MMR003002 | MMR003002028 | Ka Mawt Le (Kyaung) |
| MMR003 | MMR003D001 | MMR003002 | MMR003002041 | Naw Kaw |
| MMR003 | MMR003D001 | MMR003002 | MMR003002032 | Ko Maung |
| MMR003 | MMR003D001 | MMR003002 | MMR003002045 | Naung Kha Lone (Paingkyon Sub-township) |
| MMR003 | MMR003D001 | MMR003002 | MMR003002054 | Daw Lan (Paingkyon Sub-township) |
| MMR003 | MMR003D001 | MMR003002 | MMR003002035 | Ta Wun Hpan Ya |
